# Short-term resistance exercise inhibits neuroinflammation and attenuates neuropathological changes in 3xTg Alzheimer’s disease mice

**DOI:** 10.1186/s12974-019-1653-7

**Published:** 2020-01-03

**Authors:** Yan Liu, John Man Tak Chu, Tim Yan, Yan Zhang, Ying Chen, Raymond Chuen Chung Chang, Gordon Tin Chun Wong

**Affiliations:** 1 Department of Anaesthesiology, LKS Faculty of Medicine, The University of Hong Kong, Room K424, Queen Mary Hospital, Pokfulam, Hong Kong, SAR China; 2 Laboratory of Neurodegenerative Diseases, LKS Faculty of MedicineSchool of Biomedical Sciences, The University of Hong Kong, Hong Kong, SAR China; 3 State Key Laboratory of Brain and Cognitive Sciences, The University of Hong Kong, L4-49, Laboratory Block, Pokfulam, Hong Kong, SAR China

**Keywords:** Resistance exercise, Neuroinflammation, Alzheimer’s disease, Tau, Amyloid, Cytokines, Synapse

## Abstract

**Background:**

Both human and animal studies have shown beneficial effects of physical exercise on brain health but most tend to be based on aerobic rather than resistance type regimes. Resistance exercise has the advantage of improving both muscular and cardiovascular function, both of which can benefit the frail and the elderly. However, the neuroprotective effects of resistance training in cognitive impairment are not well characterized.

**Methods:**

We evaluated whether short-term resistant training could improve cognitive function and pathological changes in mice with pre-existing cognitive impairment. Nine-month-old 3xTg mouse underwent a resistance training protocol of climbing up a 1-m ladder with a progressively heavier weight loading.

**Results:**

Compared with sedentary counterparts, resistance training improved cognitive performance and reduced neuropathological and neuroinflammatory changes in the frontal cortex and hippocampus of mice. In line with these results, inhibition of pro-inflammatory intracellular pathways was also demonstrated.

**Conclusions:**

Short-term resistance training improved cognitive function in 3xTg mice, and conferred beneficial effects on neuroinflammation, amyloid and tau pathology, as well as synaptic plasticity. Resistance training may represent an alternative exercise strategy for delaying disease progression in Alzheimer’s disease.

## Background

Alzheimer’s disease (AD) is a chronic neurodegenerative disease that is characterized by a gradual loss of memory and cognitive function. Pathologically, accumulation of amyloid plaques and tau aggregation can be observed in several brain regions including the hippocampus and cortex [1]. Currently, there is no definitive therapeutic option for reversing AD progression, a process that involves neuroinflammation [2]. In postmortem brain of AD patients, upregulation of inflammatory cytokines and activation of microglia can readily be seen [3, 4]. Experimentally, increasing neuroinflammatory responses in the brain can exacerbate memory deficits [5], along with the exacerbation of amyloidosis and tauopathies [6]. Although therapeutic strategies targeting neuroinflammatory responses such as the use of non-steroid anti-inflammatory drugs (NSAIDs) has been shown to be effective in ameliorating cognitive deficits [7], the merits of this approach remain controversial given the known adverse effects of chronic NSAIDs treatment [8].

Exercise training has been shown to be a powerful tool in combating neuroinflammation and cognitive dysfunction in AD [9] and represents an intervention that can elicit whole body responses in metabolism and immunity [10]. Exercise training can be divided into two major types: endurance-based and resistance-based training. Each type results in different “reprogramming” of the muscle fiber to adapt to different purposes [10]. Although major studies highlight the peripheral advantages of exercise training, mounting evidence have also demonstrated beneficial effects in the central nervous system (CNS). Physical training preserves cognitive function in both human and rodents [11]. Moderate exercise training at different stages of life is associated with a reduced risk of developing cognitive impairment in the later life [12]. Cognitive performance of AD transgenic mice with excess Aβ production improved after 5 months of treadmill training [13]. An anti-neuroinflammatory effect has been proposed to underlie the neuroprotective mechanism of exercise. Exercise training can significantly reduce inflammatory cytokines and active microglia in the brains of aged or AD animals [14]. Nevertheless, most studies focused on endurance-based exercise training, while the neuroprotective effects of resistance-based training remain to be elucidated. In particular, the impact of resistance training on neuroinflammation and cognitive dysfunction deserve further clarification. We hypothesized that resistance training can suppress neuroinflammation and improve cognitive deficits. We will demonstrate our findings in an experimental AD animal model using transgenic mice with over-expression of amyloid peptide precursor, tau, and presenilin-1 (3xTg). This study aims to demonstrate the therapeutic potential of a short-term resistance training regimen especially in terms of attenuating neuroinflammation, improving synaptic function, and minimizing neuropathological changes in the brain.

## Methods

### Materials and methods

#### Animals

Nine-month-old male transgenic AD mice (B6; 129-*Psen*^*1tm1Mpm*^ Tg (APPSwe, tauP301L) 1L fa/MmJax) were obtained from the Jackson Laboratory and were held in Laboratory Animal Unit (LAU) of The University of Hong Kong, which is fully accredited by the Association for Assessment and Accreditation of Laboratory Animal Care International; 9-month-old age-matched non-transgenic mice were used as healthy controls. Mice were housed in a temperature-controlled room at 20–22 °C, humidity of 50 ± 10% and were kept on a 12/12 h light/dark cycle. All mice had access to food and water ad libitum. Mice handling and all other procedures were conducted in accordance with the National Institutes of Health guide for the care and use of laboratory animals and the Animals (Control of Experiments) Ordinance, Hong Kong, China. The use of animals was approved by the Department of Health, Hong Kong and the Committee on the Use of Live Animals in Teaching and Research, The University of Hong Kong. All efforts were made to minimize animal numbers and suffering. For transgenic mice, the experiment was conducted in two batches: each batch contains a total of 15 mice with seven in sedentary group and eight in training group. Sixteen non-transgenic mice were randomly assign to sedentary and exercise group with eight mice in each group.

### Resistance training protocol

**Fig. 1:**
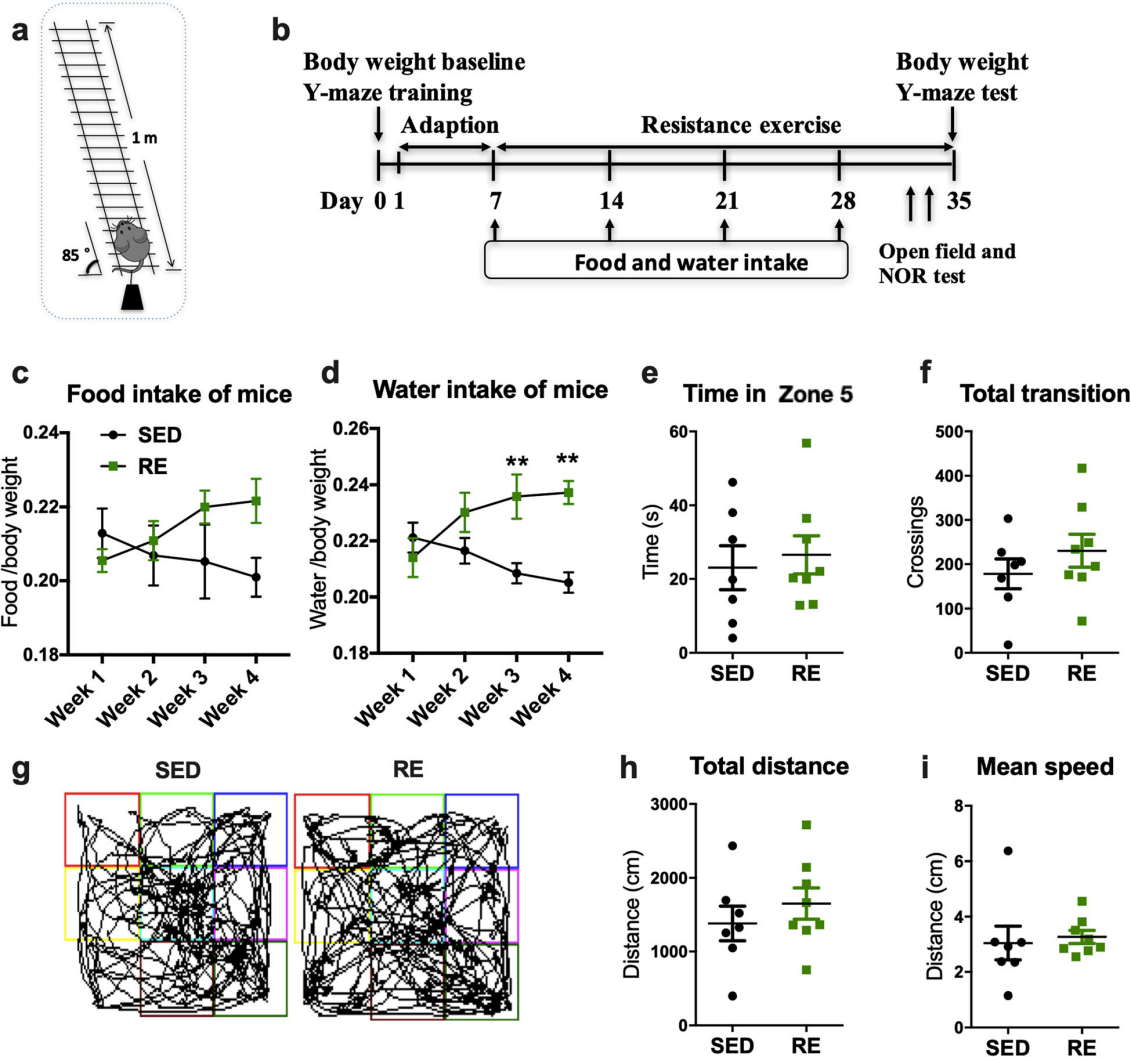
Experimental design, cognitive testing, and food and water intake. **a** A diagram of the apparatus of resistance exercise training. **b** The experimental design for resistance training and cognitive testing. **c**, **d** Food and water intake of mice. Food and water intake were measured on the first 48 h of each week. The data represented the average food/water intake of mice (normalized to the body weight) from five different cages in the exercise and sedentary groups, respectively. (two-way ANOVA, repeated measure; Bonferroni test was used for post-hoc comparisons. *n* = 5). **e**–**i** Performance in the open field test. (unpaired Student’s *t* test, *n* = 7 for the SED group and 8 for RE group, respectively). Data present as mean ± SEM, *SED* sedentary, *RE* resistance exercise

Mice were trained following a previously published protocol that uses a 1-m ladder [15] as shown in Fig. 1a. Familiarization with the exercise apparatus took place over 1 week by allowing the animals to climb up the ladder with no added resistance. Training then began on alternate days for the following 4 weeks. If the resistance training and behavior tests were at the same day, then the resistance training would be performed 2 h after the behavior test. For each training session, the mice were motivated to climb up the ladder to a total of 15 times, with progressively heavier weights attached to their tails and 2-min rest in between each climb. The weights that were loaded were equivalent to 15%, 30%, 50%, and 75% of their body weight in weeks 1, 2, 3, and 4 respectively. The intensity was carefully adjusted based on their individual difference during each exercise session.

Excessive stress was avoided during this training protocol. All the mice were trained to climb up the ladder spontaneously, only motivated with a gentle touch to the tail when they stopped in the middle of the ladder (see Additional file 1). For mice that showed symptoms of burnout (for example a failure to return to their baseline breathing pattern within 2 min or a refusal to climb), they were allowed to take extra rest time. If they still refused to climb up, the weight attached to their tail will be progressively reduced by 5 g until they resume. The reduced weight will be added back in the following trials.

### Open field test

The open field test is used to assess locomotor activity, anxiety, and depression in rodents. Mice were brought into the dim light behavior assessment room and were allowed to habituate there for 30 min. Each mouse was then gently placed in the middle of a 40 cm^2^ enclosed gridded arena and allowed to freely explore the area and 10 min of this spontaneous activity was video recorded. A video tracking software (SMART 3.0, Panlab SL) were used for data analysis. Brefily, the arena were equally divided into nine zones (as showed in Fig. 1g), a central area (zone 5) of 13.3 × 13.3 cm^2^ was demarcated and the total exploration time in this area was calculated as the parameter for anxiety/depression, and the total transition during different rooms, total distance traveled, and mean speed were documented as indicators of locomotor activity.

### Novel object recognition test

The novel object recognition (NOR) task was performed to evaluate the animals’ ability to recognize a novel object in a controlled environment. After 24 h of habituation in the open field arena in the absence of any objects, two identical sample objects (A + A) were placed at opposite but symmetrical corners of the arena. The mice were then put into the arena and allowed to explore the two objects freely for 10 min. Six hours later, one of the objects (A) was replaced by a novel object (B); the mice were placed back to the same arena with two objects (A + B). The location of the novel versus familiar object was alternated within each batch of test such that the number of times the familiar and novel object was placed at a particular corner was similar. The mice were allowed to explore the objects for 10 min and the interaction of the mouse with the objects was video recorded. An interaction was defined by the nose of the mouse pointing to the object within a distance of 2 cm. The discriminating index is the ratio of the time spent exploring the two objects (B/A) and this index was used as a measure of recognition memory.

### Y-maze test

The Y-maze test was used to examine hippocampal dependent memory function according to our previous protocol [16]. Briefly, the mice were placed in a Y-shape apparatus of which two black compartments are able to deliver electric shocks (2 Hz, 10 s, 40 ± 5 V). After being placed into the maze, electric shocks were delivered to motivate the mice to find and enter the shock-free illuminated compartment and the test is completed when it stays there for 30 s. Each mouse was trained before undergoing the exercise training protocol and the training was deemed successful if they made nine consecutive correct choices. At the end of the training period, the Y-maze test was again conducted and the number of errors and the escape latency to enter the shock-free compartment were recorded.

### mRNA extraction and real time PCR

The mice were sacrificed by CO_2_ asphyxiation and were perfused via a transcardial approach with 15 ml of cold 0.9% saline to rinse away residual blood. The brains were removed and the left hemispheres, together with the gastrocnemius muscle, were immediately immersed in precooled 4% paraformaldehyde in phosphate buffer and kept at 4 °C for further studies. The frontal cortices and hippocampi were dissected from the right hemispheres. The mRNA of the livers and brains was isolated by RNAiso plus (Takara, Japan) as previously described [16]. Isolated mRNA was dissolved in DEPC water and 1 μg of mRNA was converted to complementary DNA sequence by reversed transcription using cDNA synthesis kit according to manufactures’ protocol (Takara, Japan). cDNA expression of different inflammatory cytokines and exercise-induced factors were assessed by real-time PCR with the following primers respectively: (1) IL-1ß, forward: CCTCCTTGCCTCTGATGG, reverse: AGTGCTGCCTAATGTCCC; (2) IL-6, forward: TTCACAAGTCCGGAGAGGAG, reverse: TCCACGATTTCCCAGAGAAC; (3) TNF-α, forward: CCCCAGTCTGTATCCTTCT, reverse: ACTGTCCCAGCATCTTGT; (4) IL-10, forward: CCAAGCCTTATCGGAAATGA, reverse: TTCTCACCCAGGGAATTCAA; (5) FGF-21, forward: AGATCAGGGAGGATGGAACA, reverse: TCAAAGTGAGGCGATCCATA; 6) PGC1-α: forward: AACGATGACCCTCCTCACAC, reverse: TCTGGGGTCAGAGGAAGAGA; (7) glyceraldehyde 3-phosphate dehydrogenase (GAPDH), forward: ATTCAACGGCACAGTCAA, reverse: CTCGCTCCTGGAAGATGG.

### Milliplex cytokine assays

Protein levels of IL-1β, IL-6, IL-10, MCP-1, MIP-2, and TNF-α in the serum were measured using a customized Milliplex Mouse Cytokine Immunoassay Kit (Millipore, 2620525) with Analyzer 3.1 Luminex 200 machine (Millipore, USA). Data were analyzed using corresponding software according to the manufacturer’s instructions.

### Western blot

After being sacrificed by CO_2_ asphyxiation, the frontal cortical and hippocampal tissues dissected from the left hemispheres of each mouse were homogenized in RIPA lysis buffer (Cellsignal, Danvers, MA) supplemented with protease and phosphatase inhibitors (Roche, Berlin, German). Tissues from the right hemispheres were used to evaluate the expression levels of synaptic proteins, then the synaptosomal and cytosolic fractions were freshly prepared by using Syn-PER™ Synaptic Protein Extraction Reagent (Thermo Fisher Scientific, USA) plus protease and phosphatase inhibitors (Roche, Berlin, German), following by the manufacturer’s instructions. The levels of intracellular signal proteins and synaptic proteins expressed were evaluated by Western blot analysis.

Proteins were resolved by SDS-PAGE gel and transferred to PVDF membrane (Bio-Rad, USA). After blocking with 5% non-fat milk in TBST (0.1% tween 20 in TBS), membranes were probed with different primary antibodies (Synapsin I, Thermo Fisher Scientific, MA; Synaptotagmin 1, Synaptobrevin 1 were from Synaptic Systems, Germany; Pan-tau was from DAKO, Japan; p-tau S396, p-tau S404 were from Thermo Fisher Scientific, MA; AT180 was from Innogenetics, Belgium; GADPH was from Sigma-Aldrich, UK; PSD 95, c-Jun N-terminal kinases (JNK), p-JNK, extracellular signal-regulated kinases (ERK), p-ERK, Akt, p-Akt, Bax, and BCL-2 were from Cellsignal, MA) overnight at 4 °C, followed by respective HRP-conjugated secondary antibodies for 1 h. Protein bands were visualized by enhanced chemiluminescence (ECL) reagents (Advansta, USA) and signals were captured by ChemiDoc™ Touch Imaging System (Bio-Rad, USA). The intensities of protein bands were quantified by Image Lab™ Touch Software Version 1.2 (provided by Bio-Rad, USA).

### Immunofluorescent staining

The brains were immersed in 4% paraformaldehyde overnight at 4 °C, then immersed in 20% sucrose following by 30% sucrose for further cryoprotection. The brains were cut into 20 μm thickness frozen cross-sections for immunofluorescent (IF) staining of Iba-1 and immunohistochemical (IHC) staining; to perform morphology analysis for astrocyte, sections in 30 μm thickness were used for GFAP staining. For IF staining, sections were immersed with 5% BSA (Thermo Fisher Scientific, MA) for 60 min at room temperature to block non-specific antigens, and then were incubated with anti-Iba-1 monoclonal antibody (1:300, Wako, Japan) or anti-GFAP antibody (1:500, Sigma) overnight at 4 °C. After the sections were rinsed three times in PBS, they were incubated with fluorochrome-conjugated secondary antibodies (anti-rabbit, Alexa-fluor 488 for Iba-1, or anti-mouse, Alexa-fluor 568 for GFAP; Thermo Fisher, USA) in a cassette at room temperature for 1 h. Slight agitation was applied throughout and this was followed by incubation with 3 mM DAPI (Thermo Fisher, USA) for 15 min at room temperature. The slices were then mounted using mounting medium (DAKO, Japan). LSM700 and LSM780 confocal microscopy (Carl Zeiss, Germany) were used to examine the IBA-1 and GFAP signals, respectively, and the z-stacked signals were collected. Quantification of the images was performed using ImageJ software (National Institute of Health, USA). The morphological analysis of astrocytes was performed with the Simple Neurite Tracer plugin of ImageJ [17]. Astrocytes meeting the following criteria were included for morphology analysis (see Fig. 6d for representative examples): GFAP-stained bushy shape with a single DAPI-stained nucleus and relatively intact with no more than two truncated primary processes.

### Immunohistochemistry staining

Brain slices from four mice in each group were stained simultaneously with purified anti-β-amyloid antibody (clone 4G8, was from Biolegend, USA). Briefly, following 20 min of antigen retrieval using 88% formic acid at room temperature, the slices were incubated with 1 μg/ml of the primary antibody for 60 min at room temperature. This was followed by hematoxylin counterstaining and DAB (vector laboratories, USA) was used as chromogen for detection. The pictures of Aβ staining were captured with a Leica microscope (Leica CTR 5000; Germany). Quantification of the Aβ deposits was performed using ImageJ software.

### Statistical analysis

Statistical analyses were performed using Prism 7.0 (Graphpad Software, USA). All data were displayed as mean ± SEM. Body weights were analyzed using paired Student’s *t* test (compared to baseline). The unpaired Student’s *t* test was performed for behavioral tests, Western immunoblots, real-time PCR and IF staining, as well as IHC staining (compared to sedentary mice)**.** The food and water intake during resistance training and the process complexity of astrocytes were analyzed using two-way ANOVA (repeated measure, post-hoc test: Bonferroni test). The accepted level of significance in all cases was *p* < 0.05.

## Results

### Resistance exercise training did not induce stress in 3xTg mice

Stressed animals often display abnormal patterns of eating, drinking, and signs of anxiety. To determine whether the animals were unduly stressed by undergoing resistance training, we carefully monitored the food and water intake of mice, which were normalized to their body weight. Food intake was not affected by resistance exercise (Fig. 1c), but mice in exercise group consumed more water compared with sedentary mice from the third week onwards (Fig. 1d. Week 3: + 0.02737, *p* = 0.0063; week 4: + 0.03214, *p* = 0.0012). In addition, there was no difference in zone transition, central area duration time, total distance traveled, and mean movement speed in the open field test (Fig. 1e–i), suggesting that resistance protocol did not induce locomotor deficit or anxiety/depression in 3xTg mice. Similarly, this training protocol did not induced locomotor deficit or anxiety/depression in the wild-type mice (Additional file 2a-d).

### Resistance exercise training prevented body weight gain and preserved cognitive function in 3xTg mice

**Fig. 2:**
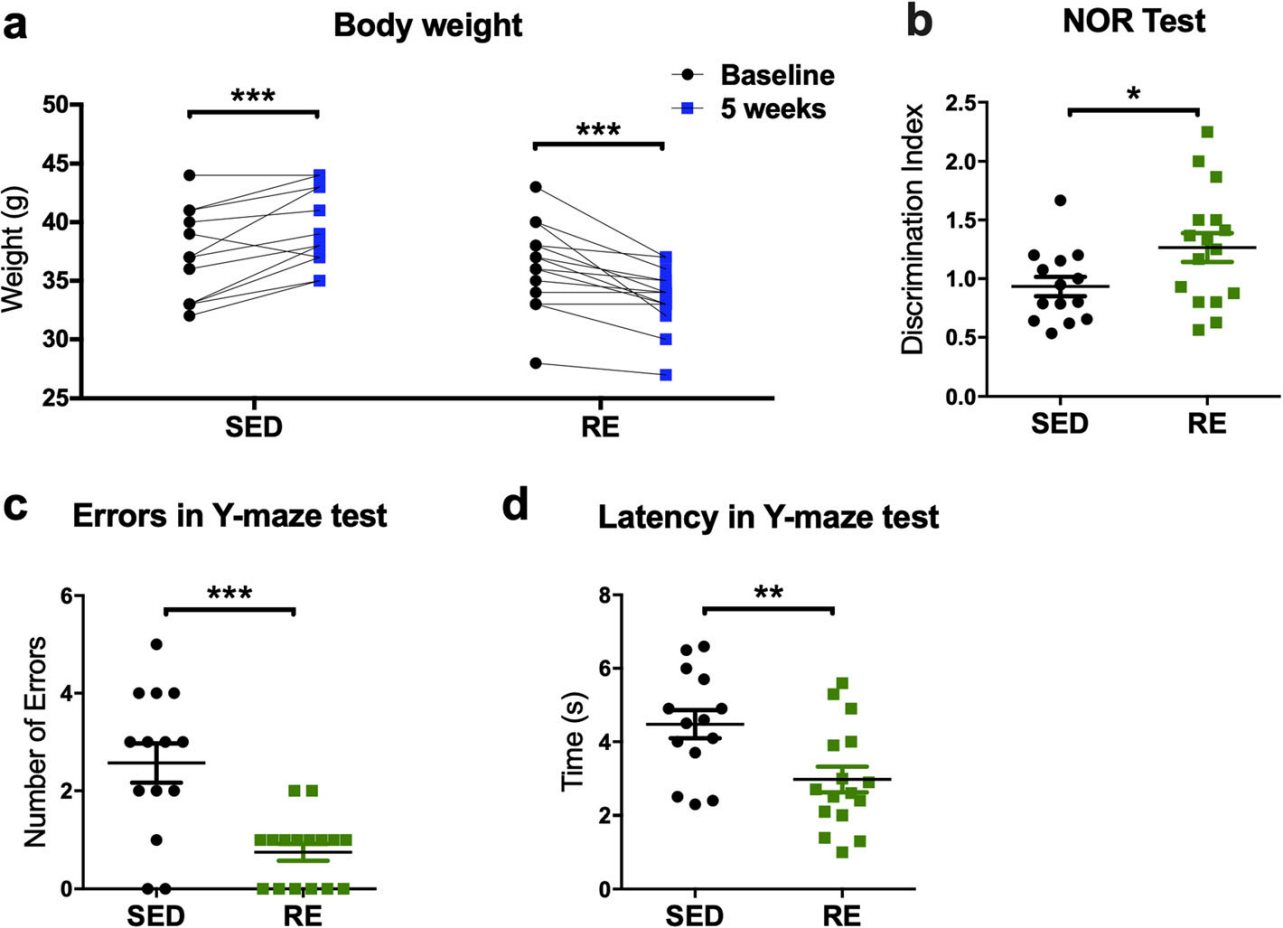
Effects of resistance exercise on body weight and cognitive performance. **a** Body weight of mice. (paired Student’s *t* test, *n* = 7 for SED group and 8 for RE group, respectively; compared to baseline). **b** Performance in the NOR test as assessed by the discrimination index. (unpaired Student’s *t* test, *n* = 14 for SED group and 16 for RE group, respectively). **c**, **d** Y-maze training and test were performed before and at the end resistance exercise training respectively. Cognitive performance was assessed by the number of error and escape latency. (unpaired Student’s *t* test, *n* = 14 for SED group and 16 for RE group, respectively). *p < 0.05, **p < 0.01, ***p < 0.001, data present as mean ± SEM. *SED* sedentary, *RE* resistance exercise

Weight gain during adulthood may be associated with significantly increased risk of major chronic diseases and decreased odds of healthy aging [18]. Weight control is a well-known effect of exercise and the body weight of the mice was monitored throughout the experimental period (outlined in Fig. 1b). After 5 weeks, the mice in the sedentary group had increases in their body weight compared to their baselines (2.286 ± 0.5286 g, *p* = 0.0008; Fig. 2a). In contrast, the body weight of mice in the exercise group was significantly decreased compared to their baselines (− 2.813 ± 0.5494 g, *p* < 0.0001; Fig. 2a), in the absence of any decrease in food or water intake or any demonstrable stress (Fig. 1c–i). Similar changes in body weight were seen in wild-type mice (SED: + 2.25 ± 0.7734, *p* = 0.0227; RE: − 3.25 ± 0.5901, *p* = 0.0009; Additional file 2e). Our results indicated that short-term resistance training reduced the body weight of middle-aged mice compared with sedentary mice. In 3xTg mice, resistance training increased recognition index in the NOR test (0.3315 ± 0.1526, *p* = 0.0384, Fig. 2b), as well as reduced the escape latency (− 1.504 ± 0.519, *p* = 0.0072, Fig. 2c) and the number of errors (− 1.821 ± 0.4174, *p* = 0.0002, Fig. 2d) in the Y-maze test. However, resistance exercise did not induce any change in the cognitive function of wild-type mice using NOR and Y maze test (Additional file 2f–h).

### Resistance exercise training increased the expression of synaptic proteins in 3xTg mice

**Fig. 3:**
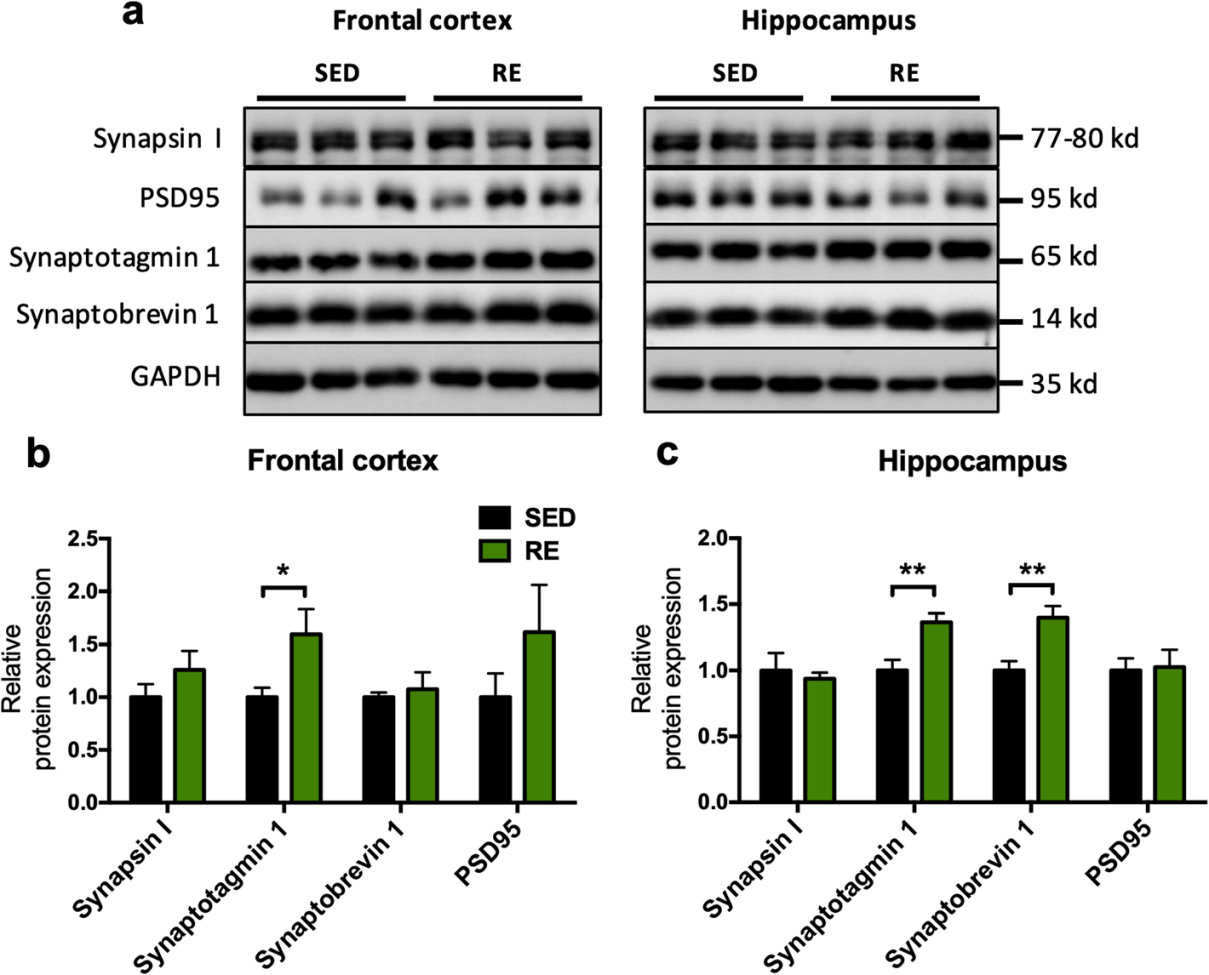
Effects of resistance exercise on expression of synaptic proteins. **a** Representative blots of Synapsin I, PSD95, Synaptotagmin 1, and Synaptobrevin 1 for frontal cortex (left) and hippocampus (right). **b**, **c** The analysis of protein expression in the frontal cortex and hippocampus. Band intensity was normalized to that of GAPDH. (unpaired Student’s *t* tests, compared to sedentary mice. *n* = 5 for SED group and *n* = 6 for RE group, respectively. *p < 0.05, **p < 0.01, data present as mean ± SEM). *SED* sedentary, *RE* resistance exercise

Synaptic dysfunction and loss of synaptic markers are early changes seen in AD [6]. Therefore, we evaluated the expression of several synaptic markers in frontal cortices and hippocampi of 3xTg mice, as these two brain regions are particularly vulnerable to neurodegenerative diseases and AD-related neuropathological [19]. Synapsin I, synaptotagmin 1, and synaptobrevin 1 are important presynaptic vesicle proteins, while PSD 95 is an important postsynaptic synaptic structure protein. We found an increase of synaptotagmin 1 from the synaptosome fraction of the frontal cortex of the active compared to the sedentary mice (0.5964 ± 0.2525, *p* = 0.0458; Fig. 3b). Similarly, the expression of synaptotagmin 1 and synaptobrevin 1 were increased in the synaptosome fraction of the hippocampi from the exercise group (synaptotagmin 1: 0.3662 ± 0.1037, *p* = 0.0064; synaptobrevin 1: 0.401 ± 0.1129, *p* = 0.0062; Fig. 3c). No difference was found in the level of synapsin I or PSD 95. This suggests that resistance exercise enhances presynaptic vesicular proteins, rather than postsynaptic structural proteins.

### Resistance exercise training reduced amyloid deposits in the brain

**Fig. 4:**
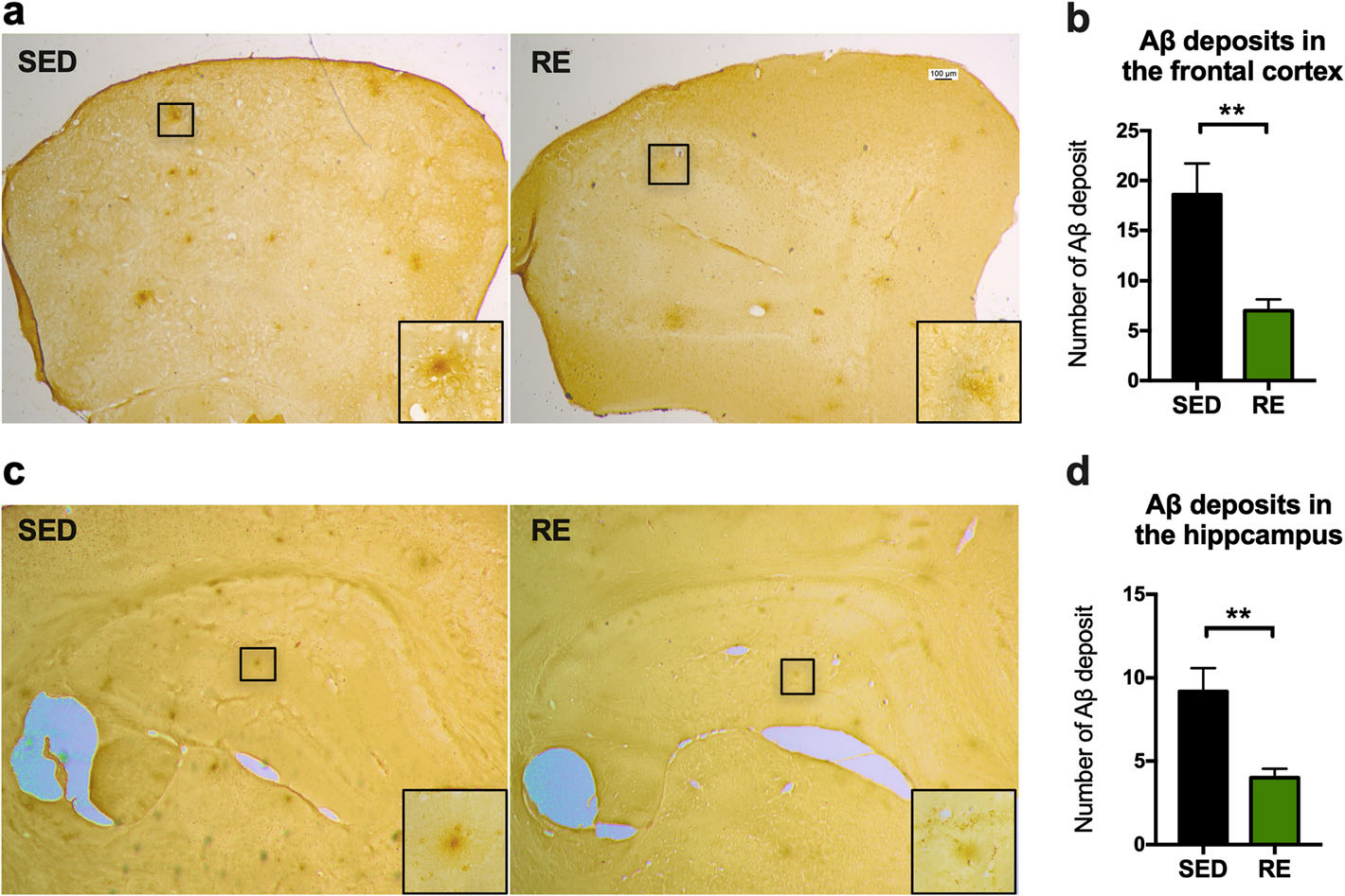
Immunochemical staining of amyloid plaques. **a**, **c** Representative images of of amyloid plaques with a plaque-specific antibody (4G8) in the frontal cortex and the hippocampus. Zoomed-in image demonstrating typical morphology of amyloid deposit were presented in the bottom right corner of each image. Scale bar: 100 μm. **b**, **d** The number of amyloid plaques was analyzed using ImageJ. (unpaired Student’s *t* tests, compared to sedentary mice, *p < 0.05, **p < 0.01. *n* = 5, data present as mean ± SEM). *SED* sedentary, *RE* resistance exercise

Amyloid plaques and hyperphosphorylated tau, pathological hallmarks of AD, can readily be seen in various brain regions of 3xTg mice. We assessed the effect of resistance exercise on the degree of amyloid deposits seen in the frontal cortex (Fig. 3a) and hippocampus (Fig. 4c). The number of amyloid plaques were significantly reduced by resistance exercise in both the frontal cortex (− 11.6 ± 3.326, *p* = 0.0082; Fig. 3b) and hippocampus (− 5.2 ± 1.497, *p* = 0.0084; Fig. 4d).

### Resistance exercise training reduced total and hyperphosphorylated tau in the brain

**Fig. 5:**
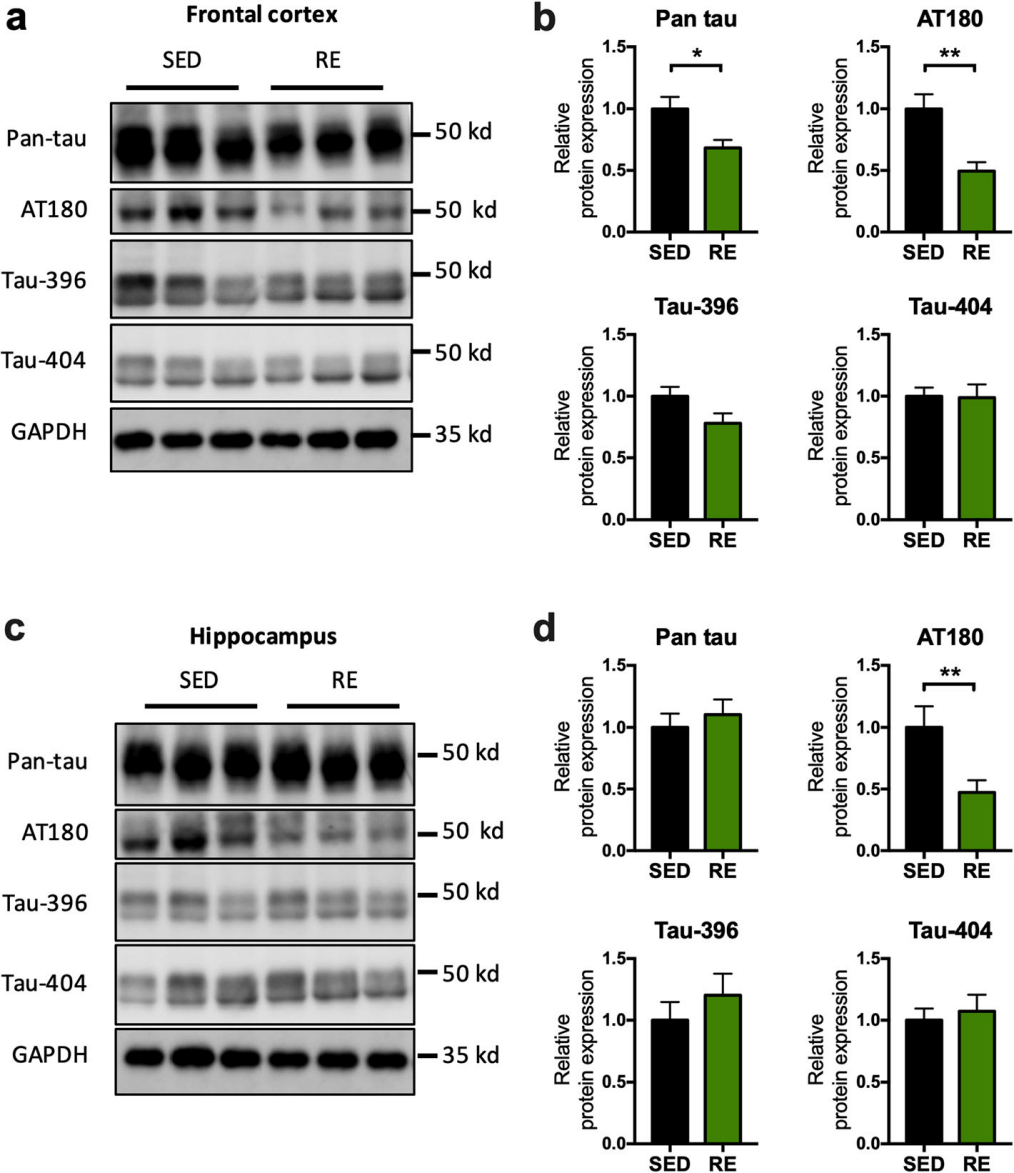
Effects of resistance exercise on tau phosphorylation. **a**, **c** Representative blots of tau protein in the brain. **b**, **d** The analysis of protein expression with the band intensity normalized to that of GAPDH. AT180 was the antibody used to detect tau phosphorylated at residue Thr231. (unpaired Student’s *t* tests, compared to sedentary mice, *p < 0.05, **p < 0.01. *n* = 6, data present as mean ± SEM). *SED* sedentary, *RE* resistance exercise

We further evaluated the effect of resistance exercise on tau phosphorylation (AT180, p-tau S396, p-tau S404) and total tau using Western blot. The levels of total tau (− 0.3168 ± 0.1142, *p* = 0.0197; Fig. 5b) and phosphorylation of tau-AT180 (− 0.5048 ± 0.1358, *p* = 0.004; Fig. 5b) were significantly decreased in the frontal cortex; the level of phosphorylation of tau-AT180 (− 0.5279 ± 0.1978, *p* = 0.0236, Fig. 5d) was also decreased in hippocampus in the exercise group. No significant changes were found for p-tau 396/404.

### Resistance exercise training reduced the activation of microglia and astrocytes in the brain

**Fig. 6:**
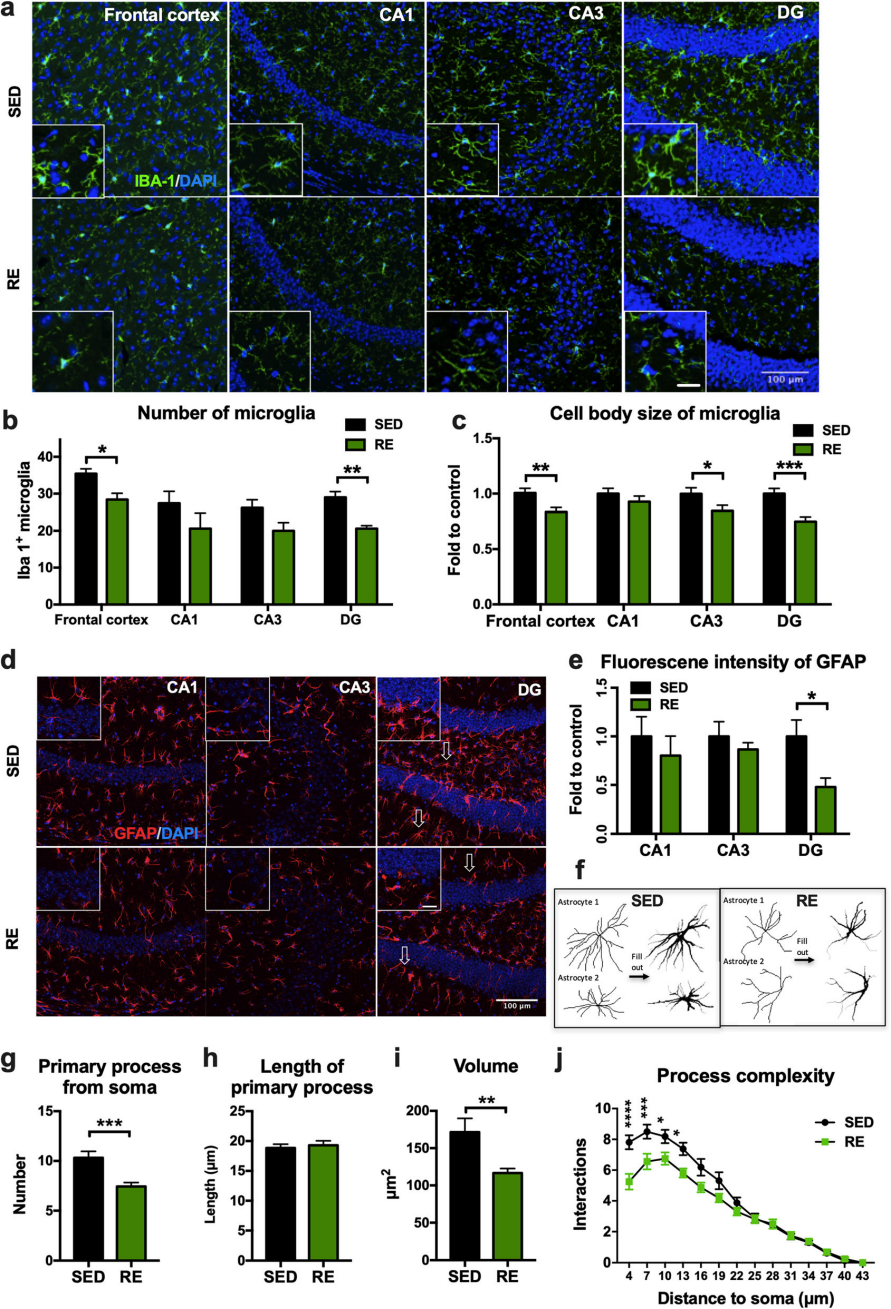
Effects of resistance exercise on microglial activation. **a** Representative confocal photographs taken with a × 20 objective lens showing the Iba-1-positive microglia (green) and DAPI (blue) in the frontal cortex and different sub-regions of hippocampus. Scale bar: 100 μm (for zoomed in images, scale bar = 20 μm). The number of microglia in each brain region was measured using ImageJ. **b** Cell count of Iba-1-positive microglia in the frontal cortex and different sub-regions of the hippocampus. **c** Cell body size of microglia in the frontal cortex and different subregions of the hippocampus. For each mouse, data represent the mean value of three brain slices. **d** Representative confocal photographs taken with a × 20 objective lens showing the GFAP positive astrocyte (red) and DAPI (blue) in the different sub-regions of hippocampus. Scale bar: 100 μm. **e** Quantification of immunofluorescent intensity of the GFAP signal. **f** Morphological analysis of astrocytes, the representative picture showing the morphological feactures of astrocytes that were marked with arrows in picture **d**. **g**–**j** The result of morphological analysis of astrocyte in DG region. (unpaired Student’s *t* test, *p < 0.05, **p < 0.01, ***p < 0.001, compared to sedentary mice. *n* = 5 and 4 for IBA-1 and GFAP staining, respectively. Data presented as mean ± SEM. *SED* sedentary, *RE* resistance exercise

After resistance training, the number of Iba-1^+^ microglia were significantly decreased in the frontal cortex and the DG region of hippocampus compared to those from sedentary mice (frontal cortex: − 7 ± 2.195, *p* = 0.0128; DG: − 8.4 ± 1.778, *p* = 0.0015; Fig. 6b) with no significant difference found in other hippocampal regions (CA1: − 6.8 ± 5.312, *p* = 0.2364; CA3: − 6.2 ± 3.072, *p* = 0.0783; Fig. 6b). We further analyzed the cell body size of microglia and demonstrated smaller cell body in @the frontal cortex, DG, and CA3 region following resistance exercise (frontal cortex: − 0.1715 ± 0.05951, *p* = 0.0042; CA1: − 0.1214 ± 0.07045, *p* = 0.0861; CA3: − 0.1549 ± 0.07683; *p* = 0.045; DG: − 0.251 ± 0.06598, *p* = 0.0002; Fig. 6c).

Reactive astrogliosis in the hippocampus was assessed by GFAP staining. After resistance exercise, the fluorescence intensity of GFAP was significantly decreased in the DG region (− 0.522 ± 0.1923, *p* = 0.0349; Fig. 6e). Further morphological analysis of the astrocytes in the DG region revealed that resistance exercise reduced the number of primary processes (− 2.875 ± 0.7675, *p* = 0.0008; Fig. 6g), volume (− 54.8 ± 19.28, *p* = 0.008; Fig. 6i), and process complexity within a radius of 4 to 13 μm from the soma (radius = 4 μm: 2.563 ± 0.4609, *p* < 0.0001; radius = 7 μm: − 1.938 ± 0.4609, *p* = 0.0004; radius = 10 μm, − 1.438 ± 0.4609, *p* = 0.0268; radius = 13 μm: − 1.563 ± 0.4609, *p* = 0.0107; Fig. 6j).

### Inflammatory factors and exercise-induced factors release following by resistance exercise training

**Fig. 7:**
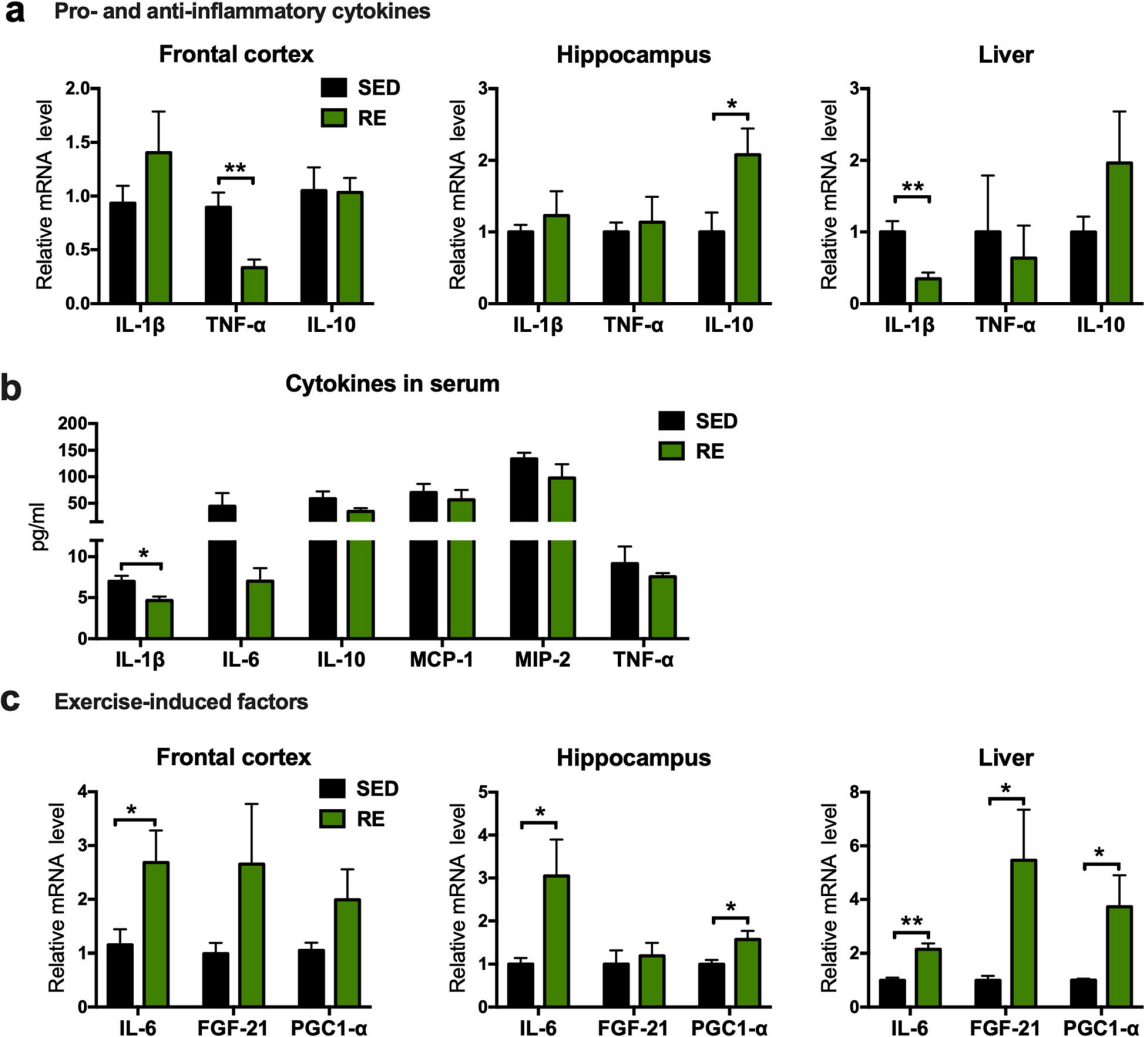
Inflammatory and exercise induced factors following resistance exercise. **a** Relative mRNA levels for various inflammatory cytokines in the brain and liver. **b** Protein levels of various inflammatory cytokines in the serum. **c** Relative mRNA levels for exercise induced factors in the brain and liver (unpaired Student’s *t* test, *p < 0.05, **p < 0.01, compared to sedentary mice. *n* = 5 for RT-PCR and 6 for Milliplex assay, data present as mean ± SEM). *SED* sedentary, *RE* resistance exercise

Activated microglia and astrocytes secrete increased levels of pro-inflammatory cytokines, such as interleukin (IL)-1β and TNF-α. Consistent with the reduction of activated microglia, we observed a significant decrease in the mRNA expression of TNF-α (− 0.5605 ± 0.1555, *p* = 0.0069; Fig. 7a) and IL-1β (− 0.6519 ± 0.1756, *p* = 0.0059; Fig. 7a) in the frontal cortex and liver respectively following resistance exercise. The anti-inflammatory mediator IL-10 was also increased in the hippocampus of the active compared to sedentary mice (1.078 ± 0.4571, *p* = 0.0461; Fig. 7a). The downregulation of pro-inflammatory markers in the frontal cortex and liver, as well as the upregulation of anti-inflammatory markers in the hippocampus suggest a peripheral as well as central anti-inflammatory effect from resistant exercise. In the serum, we detected a significant lower protein level of IL-1β in exercised mice (− 2.339 ± 0.8131, *p* = 0.0183) but no differences were seen in the expression of IL-6 (*p* = 0.1331), IL-10, MCP-1, MIP-2, and TNF-α.

The levels of a number of exercise-induced factors were changed following resistance exercise. IL-6 was increased in both the brain (FC: 1.535 ± 0.6642, *p* = 0.0496 and hippocampus: 2.049 ± 0.859, *p* = 0.0442) and the liver (1.145 ± 0.241, *p* = 0.0014, Fig. 7b). FGF-21 was also increased in the hippocampus (4.461 ± 1.899, *p* = 0.0468; Fig. 7b). PGC1-α was significantly increased in both the hippocampus (0.5686 ± 0.2238, *p* = 0.0346) and the liver (2.729 ± 1.176, *p* = 0.0489; Fig. 7b).

### Changes in intracellular signal transduction proteins in the brain following resistance exercise

**Fig. 8:**
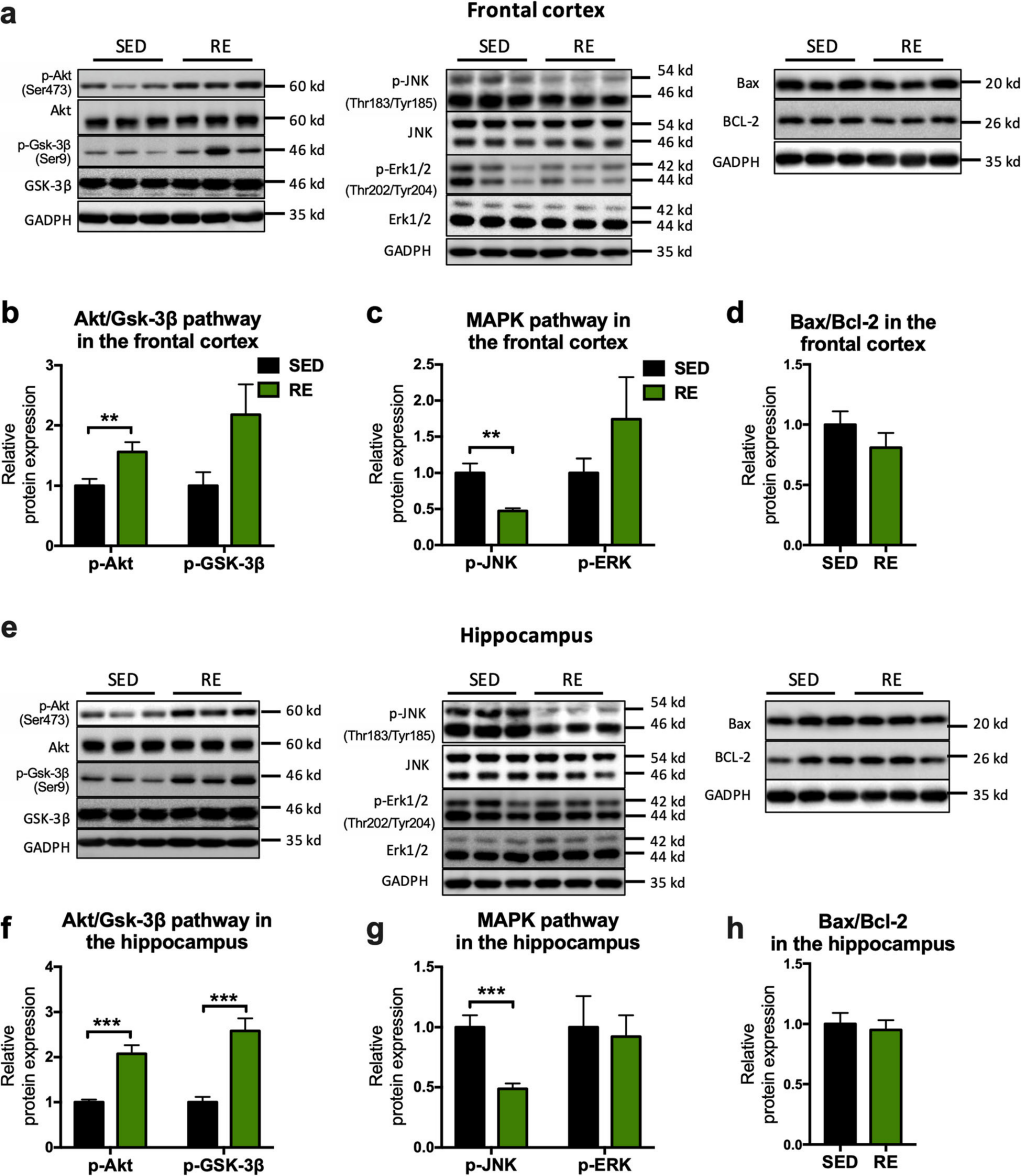
Modulation of intracellular signaling pathways after resistance exercise. **a**, **e** Representative blots of Akt, JNK, ERK, and Bax/Bcl-2 in the frontal cortex and hippocampus. **b**–**d** The analysis of protein expression in the frontal cortex. **f**–**h** The analysis of protein expression in the hippocampus. Band intensity was normalized to that of GAPDH. For Akt, GSK-3β, JNK, and ERK, the phosphorylated forms were normalized to their total forms. (unpaired Student’s *t* tests, *n* = 6, *p < 0.05, **p < 0.01, compared to sedentary mice. Data present as mean ± SEM). *SED* sedentary, *RE* resistance exercise

The above findings suggest a protective role of resistance training against cognitive impairment, synaptic dysfunction, neuropathology, and neuroinflammation. We next investigated the intracellular mechanism associated with these changes. The phosphorylated Akt (ser 473) was significantly increased by resistance training (FC: 0.5592 ± 0.1988, *p* = 0.0184; hippocampus: 1.074 ± 0.1991, *p* = 0.0003; Fig. 8b, f), along with a significant increase of GSK-3β (Ser9) in the hippocampus (FC: 1.18 ± 0.5526, *p* = 0.0586; hippocampus: 1.581 ± 0.3045, *p* = 0.0004; Fig. 8b, f); while phosphorylated JNK (Thr183/Tyr185) was significantly decreased following resistant training (FC: − 0.5258 ± 0.1369, *p* = 0.0033; hippocampus: − 0.5126 ± 0.1084, *p* = 0.0008; Fig. 8c, g). The protein levels of the regulators in Akt/GSK-3β, MAPKs, and apoptotic pathway were not changed by resistance training in wild-type mice (Additional file 3a-c).

## Discussion

Currently, there are no disease-modifying drugs available for Alzheimer’s disease and available pharmacological and supportive therapy are aimed at slowing disease progression. Clinical studies suggest physical exercise improves cognitive function and reduces the risk of developing AD [20]. The majority of the relevant studies are focused on the benefits of aerobic exercise, with only relatively few studies addressing whether resistance exercise, especially on a short-term basis, can improve learning and memory. To the best of our knowledge, short-term resistance exercise has not been previously shown to confer beneficial effects on cognitive function and neuropathology in 3xTg-AD mice and data from this study represents a novel finding.

Synaptic vesicle recycling and structural homeostasis are essential for maintaining normal cognitive function [19]. Neurotransmitters are released from synaptic nerve terminals via synaptic vesicle in response to a threshold action potential and the subsequent calcium influx. Therefore, functional improvement in the cognitive performance is likely to involve synaptic vesicle protein and synaptic structural protein changes. Indeed, we found that resistance exercise increased the expression of synaptotagmin-1 in the synaptosome fractions from the frontal cortex. Synaptotagmin-1 is a key calcium sensor for synaptic vesicle exocytosis, and have been implicated in synaptic vesicle endocytosis. Decrease in synaptotagmin-1 may lead to synaptic vesicle recycling disruption [21]. Synaptobrevin-1 was increased in the synaptosome fractions from hippocampal tissue after resistance exercise. This protein is essential for membrane fusion and decline in synaptobrevin-1 is reported to cause impaired synaptic development and induces slow neurodegeneration [22]. However, no difference was found in the levels of synapsin I or PSD 95. Thus, resistance exercise appeared to improve presynaptic vesicle recycling and synaptic transmission, rather than postsynaptic structure.

Translation of the overexpressed AD-related genes in the 3xTg mice is restricted mainly to the cerebral cortex and hippocampus, displayed as amyloid-β plaques and tau pathology, both of which are associated with malfunctioning of synapses. Aerobic exercise has been shown to reduce Aβ neurotoxicity [23] and tau pathogenesis [24] in the brain. Similar to these findings, we demonstrated a prominent reduction in amyloid deposits in the frontal cortex and hippocampus. Moreover, resistance training reduced the expression of hyperphosphorylated tau at the AT180 epitope. AT180 is a monoclonal antibody that specifically binds with tau at Thr 231 and is widely used to evaluate tau in tangles and paired helical filaments (PHFs) [25]. Combining with our result showing that resistance training decreased the expression of pan tau, these data indicated that the short-term resistance training reduced Aβ deposition, tau hypophosphorylation, and total tau burden in 3xTg mice.

Mounting evidence suggests that AD probably results from neuroinflammatory response that are triggered by accumulating amyloid plaques and neurofibrillary tangles (NFTs) [26]. This inflammatory hypothesis is supported by findings from clinical studies and animal experiments showing that anti-inflammatory agents attenuated neuronal dysfunction in AD [27, 28]. Physical exercise confers broad anti-inflammatory effects in many neurodegenerative diseases, probably by affecting the secretion of inflammatory cytokines/adipokines via the muscle-adipose crosstalk [29, 30]. It has been shown that exercise can improve the immune status of the brain, either by inhibiting the production of pro-inflammatory factors such as IL-1β [31], or by promoting the synthesis of anti-inflammatory factors such as IL-10 [32]. Interestingly, some studies demonstrate that the anti-inflammatory effect of exercise in AD was attributed to the clearance of amyloid plaques [23], the plaques being a trigger of inflammation. Activated microglia and reactive astrocytes are identified as inflammatory pathology markers in AD [33]. In this study, we showed a decrease in microglial activation in the frontal cortex and hippocampus after resistance training, accompanied by a decrease in the activation of astrocytes in the DG region of the hippocampus. Activated microglia and astrocyte are the main source of pro-inflammatory cytokines in the brain. Consistent with this, there was also a decrease in mRNA expression of TNF-α in the frontal cortex and increased mRNA expression of IL-10 in the hippocampus. Previous studies has shown that levels of TNF-α is increased in the frontal cortex [34] but remained stable in the hippocampus in 3xTg mice [35]. The distinct changes of TNF-α levels in the fontal cortex compared to the hippocampus could be due to the differential transcription of different cytokines in different brain regions. IL-10 is one of the more important anti-inflammatory cytokines. Signaling molecules including Akt and JNK has been shown to regulate the production of IL-10 [36], and the elevated expression of IL-10 in the hippocampus was consistent with our findings with the intracellular signaling pathways. However, the level of IL-10 remained unchanged in the fontal cortex, indicating that the molecular mechanisms regulating IL-10 production in the brain maybe region specific and are complex.

Systemic inflammation is well-established to contribute to the progression of AD and has been shown to predate the deposition of neurofibrillary tangles and amyloid plaques [37]. Adipocytes are important sources of peripheral inflammatory cytokines. Recent data demonstrated a direct anti-inflammatory effect of exercise by suppressing the production of several major pro-inflammatory cytokines including TNF-α and MCP-1 by adipocytes [38]. Similarly, we also identified a decreased mRNA expression and protein level of IL-1β in the liver and serum, respectively in the active mice.

Interestingly, there was also an increase in the pro-inflammatory cytokine IL-6, in both the brain and liver of the active group. IL-6 is considered different from IL-1β and TNF-α, in that the latter two are considered near-universal pro-inflammatory factors. IL-6, on the other hand, mediate multiple functions. IL-6 participates in both trans-signaling and the classic signaling pathways during the inflammatory response and both pathways induce a complex range of responses, even in absence of inflammation. It is therefore argued that IL-6 plays a role in somatic efforts in maintenance, including resistance against inflammation, pathogen tolerance, and tissue repair [39]. More importantly, in our model, resistance exercise induced IL-6 secretion is not accompanied by an elevation in IL-1β and TNF-α. Rather, resistance exercise stimulated the production of the anti-inflammatory cytokine IL-10. Through classic signaling, IL-6 upregulates lipolysis in viscera [40, 41], which might explain the significant body weight loss, attributed to mainly the reduction in adipose tissue, and the decrease in pro-inflammatory factors that were observed in the resistance exercise group. On balance, it could be argued that IL-6 release following resistance exercise is more anti-inflammatory than pro-inflammatory in its net effect.

Other than IL-6, we also evaluated the expression of two other exercise-induced cytokines, fibroblast growth factor 21 (FGF-21) and PGC-1α [42]. FGF-21 is mainly produced in the liver and can permeate into CNS through the blood-brain barrier (BBB), binding with its essential co-receptors in the brain, thereby mediating neuroprotective effects [43]. The mRNA level of FGF-21 remained unchanged both in the frontal cortex and hippocampus, which is reasonable as FGF-21 is reported to be primarily generated in peripheral organs. However, there was a remarkable increase in expression of FGF-21 in the liver. Previous study showed that peripherally derived FGF-21 leaked into the injured brain and facilitate remyelination in a mouse model of lysophosphatidylcholine-induced demyelination [44]. In addition, subcutaneously injection of recombinant human FGF-21 improved cognitive function via attenuating insulin resistance and inflammatory response in an obese mouse model [45]. Whether elevated expression of FGF-21 following our resistant training also contributed to the benefits seen in the brain should be further determined.

PGC-1α is a transcriptional co-activator that is preferentially expressed in high-energy requiring cells and is responsible for mediating oxidative metabolism, as well as mitochondrial biogenesis [46]. PGC-1α has been shown to be dramatically upregulated following both aerobic and resistance exercise, contributing to exercise-induced benefits to the CNS [47]. A recent review of the effects of PGC-1α on AD pathophysiology suggest that PGC-1α could improve oxidative stress, mitochondrial dysfunction, and insulin resistance, thereby preventing neuronal cell damage and ameliorating cognitive impairment in AD [48]. Moreover, Cheng et al. demonstrated a novel role of PGC-1α in the dendritic spine formation and maintenance in the hippocampus [49]. On the other hand, irisin, the secreted form of fibronectin type III domain containing protein 5 (FNDC5) (a PGC-1α dependent membrane-bound protein), has been demonstrated to suppress the secretion of TNF-α and MCP-1 by adipocytes [38], suggesting a direct anti-inflammatory impact of PGC-1α. The enhanced level of PGC-1α after resistance exercise we observed in the liver and hippocampus might contribute to the reduced inflammation and improved synaptic transmission, respectively. On the other hand, the SIRT1-PGC1-α axis has been shown to be associated with exercise-induced mitochondrial biogenesis, which could counteract excessive mitochondrial fission and degradation in neurodegenerative diseases [50]. Moreover, it is increasingly that mitochondrial dysfunction and neuroinflammation are interdependent lesions in AD. The direct or indirect manipulation of mitochondria through interfering with bioenergetic pathways could reduce neuroinflammation in AD [51]. Therefore, the correlation of elevated mRNA level of PGC1-α with mitochondrial biogenesis, and their relationship with neuroinflammation should be further studied using the current model.

We proceeded to investigate possible signaling pathways involved in the resistance exercise-induced beneficial effects. The activity of Akt/GSK3β and mitogen-activated protein kinases (MAPKs) were evaluated as the former is pivotal to cellular proliferation, growth, and survival in response to extracellular stimuli [52] and the latter playing a key part in regulating intracellular adaptions to various extracellular stimulation such as hyperosmosis, oxidative stress, and pro-inflammatory factors [52]. Aβ oligomer inhibits the PI3K/AKT pathway, and pharmacological activation of Akt activity reduces synaptic deficits and cognitive impairment in both 5XFAD and Aβ-induced AD mice [53]. Activities of GSK-3β, the downstream factor of Akt, is closely linked with tau hyperphosphorylation and memory dysfunction that triggered by soluble form of Amyloid-β [54]. We showed a significant activation of Akt (Ser 473) and GSK-3β (Ser 9) in both the frontal cortex and hippocampus. The combining data from ours and Ali et al.’s study suggest that resistance exercise might reduce Aβ deposits and tau pathology by increasing the activity of the Akt/GSK-3β signal pathway.

The MAPK signal pathways have been shown to be activated in vulnerable brain regions of AD patients and have been proposed as therapeutic targets for AD [55]. MAPK contains the extracellular signal-regulated kinase 1/2 (ERK1/2) pathways that are essential for cell survival and the c-Jun N-terminal kinase (JNK) pathways that are vital for mediating inflammatory response. Previous studies showed that the inhibition of JNK activation attenuated amyloid plaque, tau hyperphosphorylation and cognitive deficiency in APPswe/PS1dE9 mice [56]. Although it has been shown that activation of ERK1/2 exacerbated the AD phenotype in 5X familial mice [57], few studies demonstrated the direct effect of inhibition of ERK activity in the AD phenotype. In the present study, inhibition of JNK (Thr183/Tyr185) by resistance exercise was demonstrated in both the frontal cortex and hippocampus. However, no effect of exercise training on the activity of ERK1/2 or its downstream molecules, Bcl-2 and Bax, two well-known markers of cell death were identified. Hence, resistance exercise might protect the AD brain from neuroinflammation and subsequent neuropathology by inhibition of the JNK pathway.

## Conclusions

To conclude, the stress-free short-term resistance exercise training confers a range of beneficial effects on the brains of 3xTg-AD mice including increasing synaptic structural proteins, reducing amyloid deposit and tau pathology, as well as attenuating neuroinflammation that culminate into improving cognitive performance. The mediation of these effects is likely to involve the Akt/GSK-3β and JNK signal pathway molecules that may represent potential therapeutic targets for AD.

## Supplementary information

**Additional file 1.** The resistance exercise training protocol. The video file shows the resistance training process in action. By the fifth-week, the mouse spontaneously climbed the ladder with 75% of its body weight attached to its tail. If necessary, the mouse was motivated to complete the climb by the researcher using a gentle touch to its tail.

**Additional file 2.** Effect of resistance training on stress, bodyweight and cognition in wild type mice. (a-d) Performance in the open field test (unpaired Student’s t-test). (e) Body weight of mice (paired Student’s t-test, compared to baseline). (f) Performance in the NOR test as assessed by the discrimination index (unpaired Student’s t-test, *n* = 8). (g and h) Cognitive performance in Y-maze test as assessed by the number of error and escape latency (unpaired Student’s t-test). *n* = 8, *p < 0.05, **p < 0.01, ***p < 0.001. Data present as mean ± SEM. SED = sedentary, RE = resistance exercise.

**Additional file 3.** The effect of resistance training protocol on intracellular signaling pathways in wild type mice. (a) Representative blots of Akt, JNK, ERK and Bax/Bcl-2 in the frontal cortex (left) and hippocampus (right). (b and c) The analysis of protein expression in the frontal cortex and hippocampus. Band intensity was normalized to that of GAPDH. For Akt, GSK-3β, JNK and ERK, the phosphorylated forms were normalized to their total forms. (unpaired Student’s t-tests, *n* = 6, *p < 0.05, **p < 0.01, compared to sedentary mice. Data present as mean ± SEM). SED = sedentary, RE = resistance exercise.

## Data Availability

The datasets during and/or analyzed during the current study available from the corresponding author on reasonable request.

## Abbreviations

AD: Alzheimer’s disease
CNS: Central nervous system
ERK: Extracellular signal-regulated kinases
FGF: Fibroblast growth factor
GAPDH: Glyceraldehyde 3-phosphate dehydrogenase
IL: Interleukin
JNK: c-Jun N-terminal kinases
NFTs: Neurofibrillary tangles
NOR: Novel object recognition
PGC1-α: Peroxisome proliferator-activated receptor gamma coactivator 1-alpha
PSD95: Postsynaptic density 95
RE: Resistance exercise
SED: Sedentary
Tg: Transgenic
TNF: Tumor necrosis factor
WT: Wild type
