# Supplementary figures and images for: Short-term resistance exercise inhibits neuroinflammation and attenuates neuropathological changes in 3xTg Alzheimer’s disease mice
Source: J Neuroinflammation. 2020 Jan 3;17:4. doi: 10.1186/s12974-019-1653-7 (PMC6942350; doi:10.1186/s12974-019-1653-7)

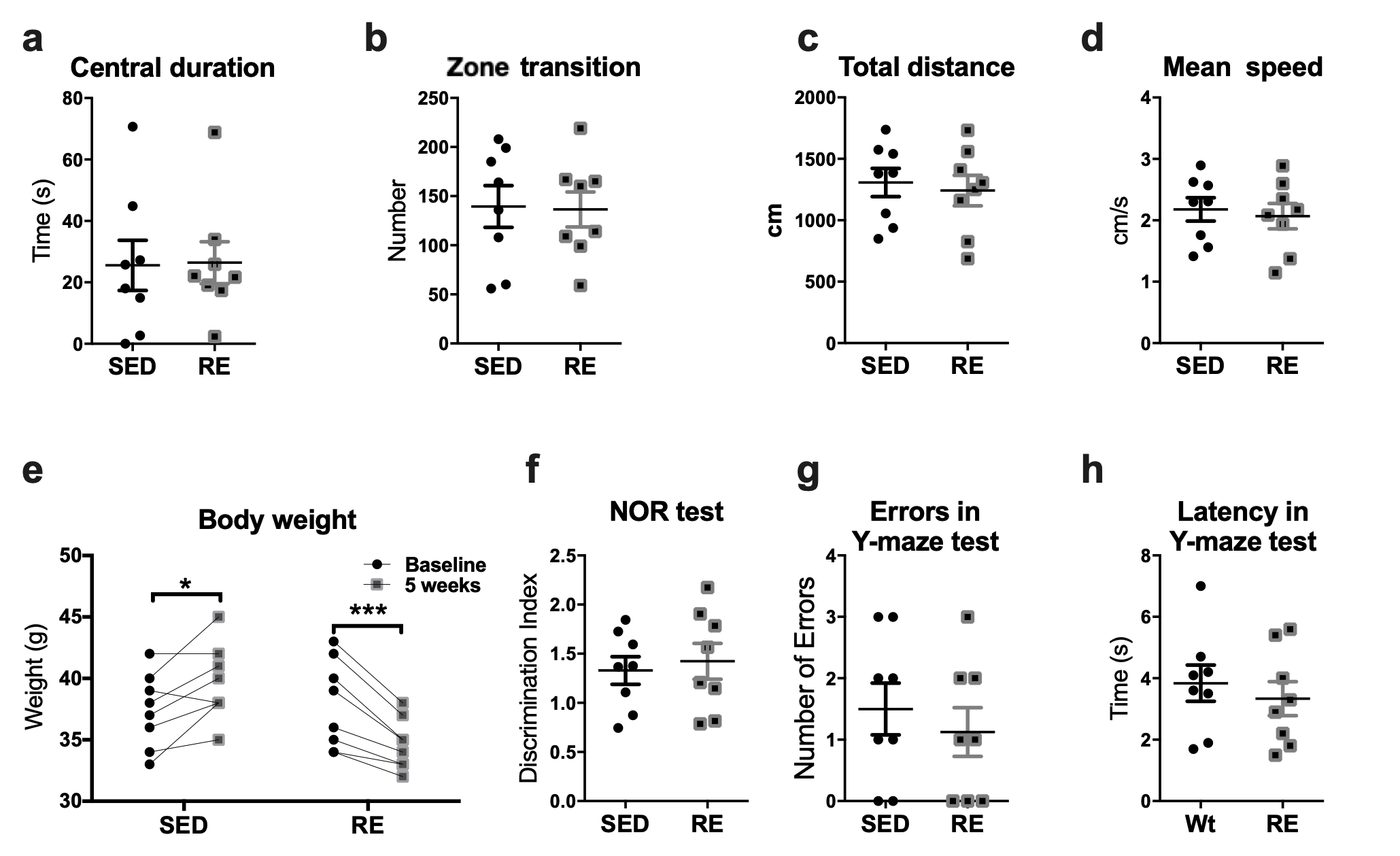

Supplement: Supplementary file 2 — Additional file 2. Effect of resistance training on stress, bodyweight and cognition in wild type mice. (a-d) Performance in the open field test (unpaired Student’s t-test). (e) Body weight of mice (paired Student’s t-test, compared to baseline). (f) Performance in the NOR test as assessed by the discrimination index (unpaired Student’s t-test, n = 8). (g and h) Cognitive performance in Y-maze test as assessed by the number of error and escape latency (unpaired Student’s t-test). n = 8, *p < 0.05, **p < 0.01, ***p < 0.001. Data present as mean ± SEM. SED = sedentary, RE = resistance exercise. [file 12974_2019_1653_MOESM2_ESM.tiff]

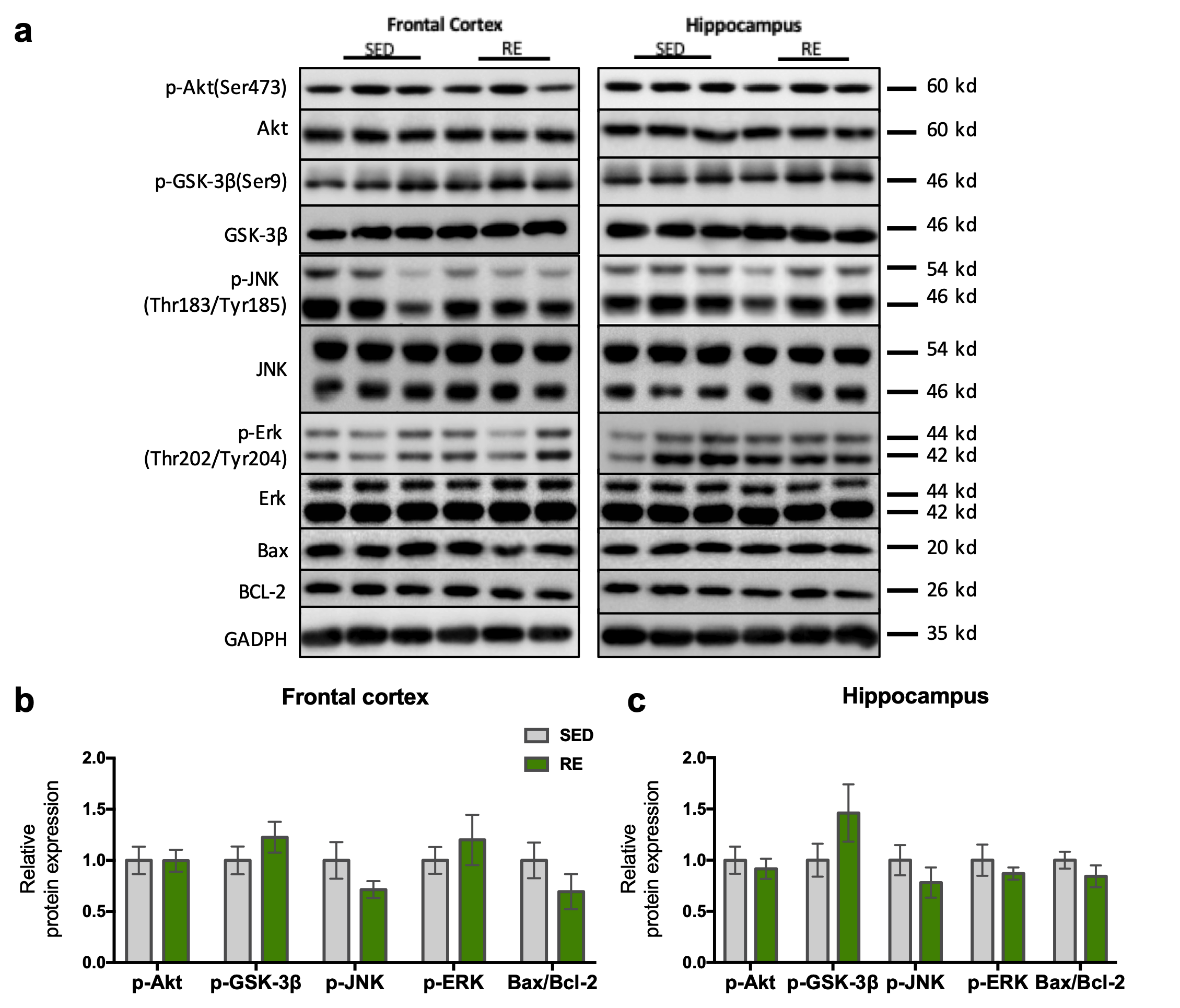

Supplement: Supplementary file 3 — Additional file 3. The effect of resistance training protocol on intracellular signaling pathways in wild type mice. (a) Representative blots of Akt, JNK, ERK and Bax/Bcl-2 in the frontal cortex (left) and hippocampus (right). (b and c) The analysis of protein expression in the frontal cortex and hippocampus. Band intensity was normalized to that of GAPDH. For Akt, GSK-3β, JNK and ERK, the phosphorylated forms were normalized to their total forms. (unpaired Student’s t-tests, n = 6, *p < 0.05, **p < 0.01, compared to sedentary mice. Data present as mean ± SEM). SED = sedentary, RE = resistance exercise. [file 12974_2019_1653_MOESM3_ESM.tiff]
